# Overexpression of a Common Wheat Gene *TaSnRK2.8* Enhances Tolerance to Drought, Salt and Low Temperature in *Arabidopsis*

**DOI:** 10.1371/journal.pone.0016041

**Published:** 2010-12-30

**Authors:** Hongying Zhang, Xinguo Mao, Chengshe Wang, Ruilian Jing

**Affiliations:** 1 College of Agronomy, Northwest A & F University, Yangling, Shaanxi, China; 2 National Key Facility for Crop Gene Resources and Genetic Improvement, Institute of Crop Science, Chinese Academy of Agricultural Sciences, Beijing, China; Purdue University, United States of America

## Abstract

Drought, salinity and low temperatures are major factors limiting crop productivity and quality. Sucrose non-fermenting1-related protein kinase 2 (SnRK2) plays a key role in abiotic stress signaling in plants. In this study, *TaSnRK2.8*, a SnRK2 member in wheat, was cloned and its functions under multi-stress conditions were characterized. Subcellular localization showed the presence of TaSnRK2.8 in the cell membrane, cytoplasm and nucleus. Expression pattern analyses in wheat revealed that *TaSnRK2.8* was involved in response to PEG, NaCl and cold stresses, and possibly participates in ABA-dependent signal transduction pathways. To investigate its role under various environmental stresses, *TaSnRK2.8* was transferred to *Arabidopsis* under control of the CaMV-35S promoter. Overexpression of *TaSnRK2.8* resulted in enhanced tolerance to drought, salt and cold stresses, further confirmed by longer primary roots and various physiological characteristics, including higher relative water content, strengthened cell membrane stability, significantly lower osmotic potential, more chlorophyll content, and enhanced PSII activity. Meanwhile, *TaSnRK2.8* plants had significantly lower total soluble sugar levels under normal growing conditions, suggesting that *TaSnRK2.8* might be involved in carbohydrate metabolism. Moreover, the transcript levels of ABA biosynthesis (*ABA1, ABA2*), ABA signaling (*ABI3, ABI4, ABI5*), stress-responsive genes, including two ABA-dependent genes (*RD20A, RD29B*) and three ABA-independent genes (*CBF1, CBF2, CBF3*), were generally higher in *TaSnRK2.8* plants than in WT/*GFP* controls under normal/stress conditions. Our results suggest that *TaSnRK2.8* may act as a regulatory factor involved in a multiple stress response pathways.

## Introduction

Plant growth and crop production are adversely affected by environmental stresses such as extreme temperatures, drought, and high salinity. As adaptable organisms, plants have developed complex signaling networks to regulate biochemical and physiological acclimation [1], [2]. Many studies have indicated the involvement of stress signaling cascades composed of second messengers [3], phospholipids [4], phosphatases [5] and protein kinases [6]. However, most of the components that transduce stress signals remain to be discovered and elucidated.

One of the major pathways by which extracellular stimuli are transduced into intracellular responses is the calcium-dependent protein kinase (CDPK) signaling cascade, which is activated by ABA and other diverse stress signals [7], [8]. CDPK kinases are calcium-regulated and are distinguished by a structural arrangement in which a calmodulin-like regulatory domain is located at the C-terminal end of the enzyme. The sucrose non-fermenting1 (SNF1) protein kinase family, belonging to the CDPK-SnRK superfamily [9], comprises SNF1 in yeast, AMP-activated protein kinases (AMPK) in mammals, and SNF1-related protein kinases (SnRKs) in plants. In yeast and mammals, all these kinases are involved in regulation of carbon metabolism and energy status in their respective systems, whereas in plants, they may represent interfaces between metabolic and stress signaling. Furthermore, accumulating evidence indicates that plant SnRKs may be hubs within a network of interacting signaling pathways, rather than being components of simple signaling cascades [10], [11].

Plant SnRKs are grouped into three subfamilies: SnRK1, SnRK2 and SnRK3. SnRK1 kinase is well characterized at the molecular and biochemical levels, and evidence indicates that SnRK1s have a role in global regulation of carbon and nitrogen metabolism, whereas SnRK2 and SnRK3 mainly function in stress signaling [12]. In the process of plant evolution, a number of duplicated protein kinases involved in plant development may have evolved as resistance genes by selection or acquisition to address various environmental stresses [13], [14]. Interestingly, recent studies have suggested that SnRK2 and SnRK3 originated by gene duplication of SnRK1, and then diverged rapidly during plant evolution to fulfill new roles that enabled plants to develop networks linking stress and ABA signaling with metabolic signaling [11]. The SnRK2 and SnRK3 gene subfamilies are unique to plants [15]. To date, most studies on SnRK2 and SnRK3 kinases focus on their involvement in response to stresses. One of best studied kinases in the SnRK3 family, *SOS2*, which is required for Na^+^ and K^+^ homeostasis and abiotic stress tolerance is involved in responses to salt stress and ABA signaling [16], [17].

Increasing evidence shows that SnRK2 genes play crucial roles in abiotic stress response, and might be involved in diverse developmental processes in plants. In *Arabidopsis*, ten *SnRK2s* have been identified, of which five members (*SnRK2.2*, *SnRK2.3*, *SnRK2.6*, *SnRK2.7* and *SnRK2.8*) are activated by ABA, and all members, except *SnRK2.9*, could be activated by hyperosmotic and salinity stresses, whereas none was activated by cold stress [18], [19]. Furthermore, overexpression of *SnRK2.8* resulted in up-regulation of stress-related genes and led to enhanced drought tolerance in *Arabidopsis* [20]. Recently, three members *SRK2D/E/I* were identified to function as main positive regulators of ABA signaling in response to water stress [21]. Similarly, 10 SnRK2s, designated *SAPK1-10*, were identified in rice. All were activated by hyperosmotic stress, and *SAPK8-10* were also activated by ABA [22]. Overexpression of *SAPK4* significantly enhanced tolerance to salt in rice [23]. Recently, ten maize SnRK2 members were cloned, and most *ZmSnRK2s* were induced by one or more abiotic stresses [24]. In wheat, the first SnRK2 cDNA clone, *PKABA1*, was isolated from an ABA-treated wheat embryo cDNA library [25]. In our recent study, the wheat *TaSnRK2.4* gene, expressed strongly in booting spindles compared to leaves, roots and spikes, was induced by multi-stresses and ABA application. Overexpression of *TaSnRK2.4* resulted in delayed seedling establishment, longer primary roots and enhanced tolerance to abiotic stresses in *Arabidopsis* [26]. Although various studies show that SnRK2s are activated rapidly in response to abiotic stresses, knowledge of specific functions of SnRK2s in wheat is fragmentary.

As a world staple crop, wheat production is constrained by muti-environmental stresses, such as drought, salinity and extreme temperatures. Therefore, understanding the molecular basis of abiotic stress responses is necessary for genetic improvement of stress resistance in wheat. In this study, we characterized *TaSnRK2.8* in wheat and observed its expression patterns under various abiotic stresses and in different wheat tissues. Abiotic stress tolerance assays indicated that *TaSnRK2.8* overexpressing in *Arabidopsis* significantly increased tolerance to drought, salt and cold stresses.

## Materials and Methods

### Plant materials, growth conditions, and stress treatments

Common wheat (*Triticum aestivum* L.) genotype “Hanxuan 10” with a conspicuous drought-tolerant phenotype was used in this study. Wheat seedling growth conditions, and stress treatment assays were performed as described previously [26]. To study the expression of the target gene at different developmental stages, wheat seedling leaves and roots, spindle leaves at booting, and spikes at the heading stage were sampled. Seedlings were grown in the growth chamber, and spindle leaves at jointing and spikes were sampled from field plots without environmental stress [26].

### Cloning the full-length *TaSnRK2.8* cDNA and sequence analysis

Various wheat tissues were collected to extract total RNA with TRIZOL reagent (Invitrogen). Based on the candidate EST of *TaSnRK2.8* from the dehydration-inducible cDNA library of wheat established in our laboratory [27], the putative full-length *TaSnRK2.8* cDNA was obtained by *in silico* cloning, and a pair of gene-specific primers was designed to amplify the full-length cDNA (F: 5'-GGGGAAACCGAGCCCTATC-3', R: 5'-CAAGTTCAGTCACAGGTTCACACATTA-3').

Database searches were performed through NCBI/GenBank/Blast. Sequence alignments and comparisons were performed using the MegAlign program in DNAStar. Protein predictions were identified using PROSITE (http://expasy.hcuge.ch/sprot/prosite.html) and SignalP (http://genome.cbs.dtu.dk/services/SignalP). ClustalW and PHYLIP were used to construct a phylogenetic tree of TaSnRK2.8, TaSnRK2.4, PKABA1 and SnRK2 members from other plant species.

### Subcellular localization of TaSnRK2.8 protein

The ORF of *TaSnRK2.8* without the termination codon was amplified with primers F: 5'-GAGA***AAGCTT***AGCCCTATCGGCCGCG -3' (Hin*dIII* site in bold italics) and R: 5'-GAGA***GTCGAC***CGCATACACGATCTCTCCACTG -3' (*Bam*HI site in bold italics), then inserted into the pJIT163-*GFP* vector. The fusion construct (*TaSnRK2.8-GFP*) and control (*GFP*) were transformed into living onion epidermal cells by particle bombardment with a GeneGun (Biorad Helios^TM^) according to the instruction manual (helium pressure, 150–300 psi). After incubation on Murashige and Skoog (MS) medium (pH 5.7) solidified with 1.5% agar at 28°C for 36–48 h, the onion cells were observed with a laser scanning confocal microscope (Leica TCS-NT).

### Expression patterns of *TaSnRK2.8* in wheat

Quantitative real-time PCR (qRT-PCR) were used to determine the expression patterns of *TaSnRK2.8*. The *Tubulin* transcript was used as an internal control to quantify the relative transcript levels. The qRT-PCR were performed in triplicate with an ABI PRISM® 7000 system using the SYBR Green PCR master mix kit (Applied Biosystems). Specific primers (F: 5'-CGGGGAGAAGATAGACGAGAATG-3', R: 5'-CTCAAAAAGCTCACCACCAGATG-3') were designed according to the cDNA sequence. The relative level of gene expression was detected using the 2^−ΔΔCT^ method [28]. ΔΔ*C*T = (*C*_T, Target_ − *C*_T, Tubulin_) _Time x_ − (*C*_T, Target_ − *C*_T, Tubuli*n*_) _Time 0_. The *C*_T_ (cycle threshold) values for both the target and internal control genes were the means of the triplicate independent PCRs. Time x is any treatment time point (1, 3, 6, 12, 24, 48 or 72 h) and time 0 represents the untreated time (0 h).

Leaves, roots, stems, and spikes were sampled to detect the transcription level of *TaSnRK2.8* in different wheat tissues. The expression of *TaSnRK2.8* in seedling leaves was regarded as standard because of its lowest expression level in that tissue, and the corresponding formula was modified as ΔΔ*C*T = (*C*_T, Target_ − *C*_T, Tubulin_) _DST_ − (*C*_T, Target_ − *C*_T, Tubulin_) _SL_. DST refers to the developmental stage tissue. In addition, to identify the relative expression of *TaSnRK2.8* in transgenic *Arabidopsis*, the *Actin* transcript of *Arabidopsis* was used to quantify the expression levels, and the lowest expression level among transgenic lines was regarded as standard.

### Transgenic plant generation

*TaSnRK2.8* cDNA containing the entire ORF was cloned into the *Kpn* I and *Sal* I sites of pPZP211 [29] as a *GFP*-fused fragment under control of the CaMV 35S promoter and NOS terminator, using primers 5'-GAGA***GGTACC***AGCCCTATCGGCCGCG-3' (*Kpn* I site in bold italics) and 5'-GAGA***GTCGAC***ATGGCTCACATCGCATACACG-3' (*Sal* I site in bold italics). The p35S-*TaSnRK2.8*-*GFP*-NOS construct and the p35S-*GFP*-NOS vector were each introduced into *Agrobacterium*, and then transferred into wild type *Arabidopsis* (Columbia ecotype) plants by floral infiltration. Positive transgenic plants over-expressing *TaSnRK2.8*/*GFP* gene (*TaSnRK2.8*/*GFP* plants) were firstly screened on kanamycin plates and then identified by RT-PCR and fluorescence detection of GFP.

### Morphological characterization of transgenic *Arabidopsis*

*Arabidopsis* seeds were sterilized for 10 min in 10% (v/v) sodium hypochlorite solution containing 0.02% (v/v) Triton X-100, rinsed with sterilized water several times, and then sown on MS medium solidified with 0.8% agar. Seeds were vernalized overnight at 4°C before transferred to a growth chamber (22°C, 70% humidity, 150 µM m^−2^ s^−1^, 12 h light/12 h dark cycle). To examine the root morphology, 3-day-old seedlings were grown on MS medium solidified with 1.0% agar, and the plates were placed vertically so that the root tips pointed downwards. To characterize seedling size and seed production of *TaSnRK2.8 Arabidopsis*, 7-day-old seedlings were planted in soil and cultured in a growth chamber.

### Total soluble sugar analyses

Total soluble sugars were determined as fructose equivalents using the anthrone colorimetric assay [30] at 620 nm with a spectrophotometer (LG-721, BioRad). After detaching from roots, four-week-old plants were put into liquid nitrogen immediately and dehydrated in a refrigerated-vacuum evaporator at 8.1 kPa air pressure and −60°C for 24 h. After dehydration, samples were dried at 80°C until a constant dry weight. Extractions were performed with 0.1 g dry material for each sample with three replications. Samples were boiled in 4 ml ddH_2_O for 4 min. After filtration the extracted filtrates were transferred to volumetric flasks (5 ml) and brought to 5 ml by addition of ddH_2_O.

### Physiological assays

For relative water content (WRA) measurements, ten 4-week-old plants with similar size in each line were detached from their roots, immediately weighed (fresh weight, FW) and then left on the laboratory bench (humidity, 45–50%, 20–22°C) until there were no further losses in weight (desiccated weight). The proportions of fresh weight loss were calculated relative to the initial plant weights. The plants were finally oven-dried to a constant dry weight (DW) for 24 h at 80°C. WRA were measured according to the formula: WRA (%) = (desiccated weight − DW)/(FW − DW) ×100.

For osmotic potential (OP) analysis, 10 similar sized 4-week-old plants in each line were collected as a sample. OP was measured with a Micro-Osmometer (Fiske® Model 210, Fiske® Associates).

Free proline was extracted and quantified from fresh leaves of well-watered seedlings (0.5 g) according to the ninhydrin-based colorimetric assay [31].

Plant cell membrane stability (CMS) was determined with a conductivity meter (DDS-1, YSI), CMS (%) = (1− initial electrical conductivity/electrical conductivity after boiling) ×100. Ten 7-day-old seedlings (grown on 1× MS medium, 0.8% agar) were placed on filter papers saturated with NaCl (300 mM) solution. When signs of stress began to appear on WT plants, seedlings were removed and immediately thoroughly rinsed with double distilled water (ddH_2_O) prior to immersion in 20 mL ddH_2_O at room temperature. After 2 h initial conductivities of the solutions were recorded. The samples were then boiled for 30 min, cooled to room temperature, and the final conductivities were measured.

Leaf chlorophyll content (SPAD value) was measured using a Minolta Chlorophyll Meter (SPAD-502) according to the Instruction Manual. SPAD values provide an indication of the relative amounts of total chlorophyll present in plant leaves, the arbitrary SPAD value can be translated to an actual value of total chlorophyll per unit area (mg/cm^2^) using the equation: chlorophyll content = SPAD value ×0.003–0.048 [32]. Ten 4-week-old plants of each line with similar size were selected to measure chlorophyll content.

Chlorophyll florescence was measured with a portable photosynthesis system (LI-COR LI-6400 XTR), and the maximum efficiencies of PSII photochemistry, Fv/Fm = (Fm-F0)/Fm, were used to assess changes in the primary photochemical reactions of the photosynthetic potential. Ten similar sized 4-week-old plants from each line were selected to determine chlorophyll fluorescence parameters. After a three-week water-withholding period, the plants were well irrigated with NaCl solution (300 mM). Chlorophyll florescence was measured before stress and 12, 24, and 36 h after stress.

Stomatal conductances were measured before stress and 12, 24 and 36 h after stress with a steady state diffusion leaf porometer (Model SC-1, Decagon). Similar sized mature rosette leaves were selected for stomatal conductance determinations and the area around the merging point of the leaf transverse midline and vein was chosen as the measuring region.

### Abiotic stress tolerance assays

WT and transgenic seeds were germinated on MS medium solidified with 0.8% agar. Seven-day-old seedlings, were planted on a sieve-like plate containing mixed soil (vermiculite∶humus = 1∶1) and cultured normally in the greenhouse. The plants were exposed to various stresses at designated time points. For drought tolerance assays, seedlings were cultured in a greenhouse (22°C, 70% humidity, 150 µM m^−2^ s^−1^, 12 h light/12 h dark cycle) without watering until phenotypic differences were evident between transgenic plants and controls, and then re-watered. For salt stress assays, *Arabidopsis* seedlings were cultured as described above. Water was withheld and then plants were well irrigated with NaCl solution (300 mM) applied from the bottom of the plate. For cold stress, plants were transferred to a 4°C growth chamber after culturing under normal conditions for three days. Further freezing tolerance assays were carried out on seedlings. Normally cultured *Arabidopsis* seedlings (4-week-old) were stressed in a −2°C, −6°C and −10°C freezer for 2 h, and subsequently cultured under normal growing conditions. Survival rates were scored after one week. All abiotic stresses tolerance experiments were triplicated.

### Gene expression analysis

10-day-old *Arabidopsis* seedlings grown on MS medium were treated, or not treated, with NaCl solution (200 mM). The seedlings were harvested 3 h after stress. Real-time RT-PCR was performed as described above, and the *Actin* gene was used as an internal control to normalize all data. The oligonucleotide primers, used for evaluating the transcript levels of *ABA1, ABA2, ABI1, ABI2, ABI3, ABI4, ABI5, RD20A, RD29B, CBF1, CBF2, CBF3, COR15A*, in the real-time RT-PCR experiments, were applied as Ding *et al.* [33].

## Results

### Isolation and sequence analysis of *TaSnRK2.8*

**Figure 1:**
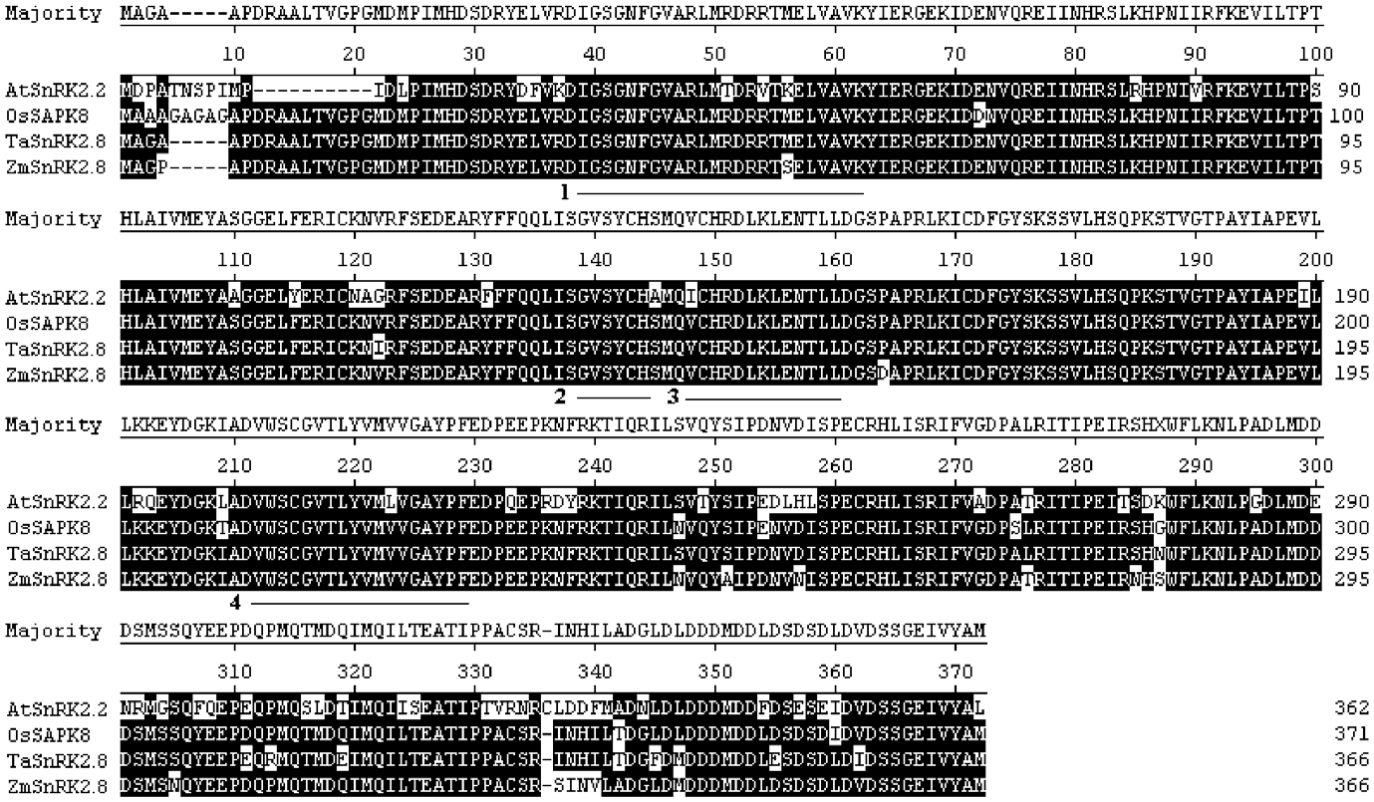
Alignment of the predicted amino acid sequences of TaSnRK2.8 and closely related SnRK2s from other plant species. The conserved prosite motifs are underlined. Regions 1–4 represent the ATP binding site, N-myristoylation site, protein kinase activating signature and transmembrane spanning region, respectively. Alignments were performed using the Megalign program of DNAStar. Common identical amino acid residues are shown in black background. Dashed lines represent gaps introduced to maximize alignment. Abbreviations on the left side of each sequence: Os, *O. sativa*; At, *A. thalian*a; Zm, *Z. mays*.

The *TaSnRK2.8* cDNA was 1431 bp in length, consisting of a 99 bp of 5' untranslated region, a 1101 bp open reading frame (ORF), and a 235 bp 3' untranslated region. The ORF encodes 366 deduced amino acid residues (AAR) with a calculated molecular mass of 42 kDa and a predicted pI of 4.87. Using a BLASTN search of the NCBI database, the deduced amino acid sequence showed homology with counterpart SnRK2 family members from other plant species, viz. *Oriza sativa, Zea mays* and *Arabidopsis thaliana* (Figure 1). TaSnRK2.8 has 94.8% identity to OsSAPK8, 94% to ZmSAPK8, and 76.5% to AtSnRK2.2, respectively. Scansite analysis indicated that *TaSnRK2.8* has potential serine/threonine protein kinase activities, and like other SnRK2 family members, TaSnRK2.8 showed a two domain structure, characterized by an N-terminus catalytic domain close to the SNF1/AMP kinase region and a regulatory C-terminus region in which a stretch of acidic amino acids forms a negatively charged domain. The N-terminal catalytic domain (28–284 AAR) is highly conserved, containing an ATP binding site (34–57 AAR) and a protein kinase activating signature (143–155 AAR). Additionally, in the catalytic domain one potential N-myristoylation site MYRISTYL (134–139 AAR) and one potential transmembrane spanning region (207–224 AAR) were found. The relatively short C-terminal domain is abundant in Asp (D), and is predicted to be a coiled-coil. Compelling evidence indicated that the C-terminal domain might have a role in activation of the kinase [34]–[36], and function in protein-protein interactions mainly involved in ABA responsiveness, and possibly involved in ABA signal transmission [22].

### Phylogenetic analysis

**Figure 2:**
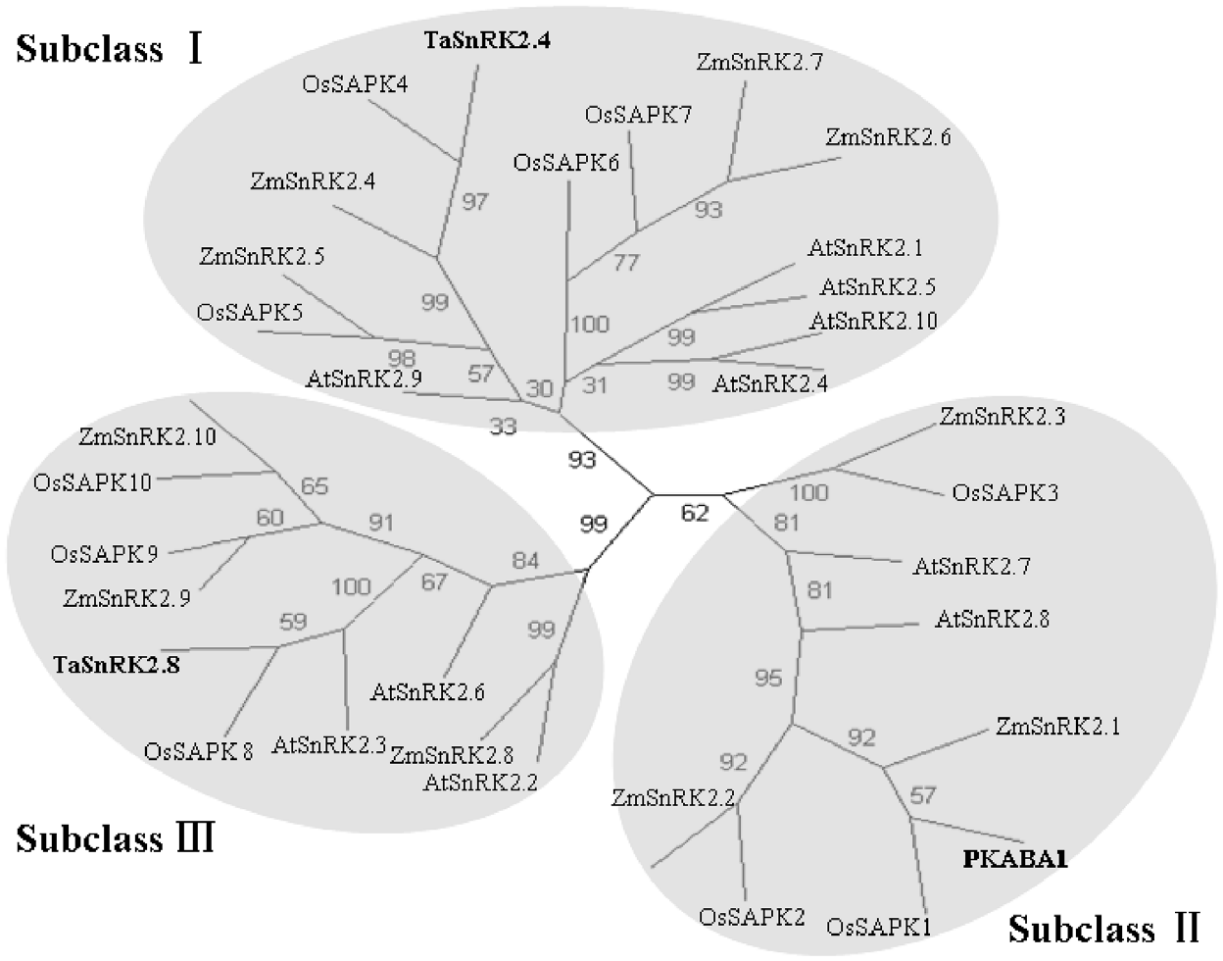
Phylogenetic tree of TaSnRK2.8 and SnRK2s from other plant species. The phylogenetic tree was constructed using the putative amino acid sequences. Three distinct isoform groups are presented in grey. The phylogenetic tree was constructed with the PHYLIP 3.68 package; Bootstrap values are in percentages.

A phylogenetic tree was constructed with the putative amino acid sequences of TaSnRK2.4, TaSnRK2.8 and all members of the rice, maize and *Arabidopsis* SnRK2 family. As shown in Figure 2, all of the SnRK2 genes could be divided into three distinct groups, consistent with previous reports [10], [22]. TaSnRK2.8 and its counterparts, OsSAPK8, ZmSAPK8 and AtSnRK2.2, were clustered in the same clade, subclass III, whereas PKABA1 and TaSnRK2.4 belong to subclass I and subclass II, respectively.

### Subcelluar localization of the TaSnRK2.8 protein

**Figure 3:**
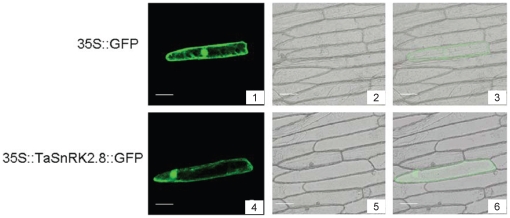
Subcellular localization of TaSnRK2.8 in onion epidermal cells. Cells were bombarded with constructs carrying *GFP* or *TaSnRK2.8-GFP* as described in materials and methods. GFP and TaSnRK2.8-GFP fusion proteins were transiently expressed under control of the CaMV 35S promoter in onion epidermal cells and observed with a laser scanning confocal microscope. Images were taken in dark field for green fluorescence (1, 4). The cell outline (2, 5) and the combination (3, 6) were photographed in bright field. Scale bar = 100 µm. Each construct was bombarded into at least 30 onion epidermal cells.

The deduced amino acid sequence contains a putative N-myristylation site and a transmembrane region, suggesting that TaSnRK2.8 might interact with the cell-membrane and nuclear system. We used onion epidermis to determine subcellular localizations of TaSnRK2.8 in living cells. The fusion construct (TaSnRK2.8::GFP) driven by the CaMV 35S promoter was transiently expressed in living onion epidermal cells. As predicted, TaSnRK2.8-GFP was present in the cell membrane, cytoplasm and nucleus (Figure 3).

### Expression patterns of *TaSnRK2.8* in wheat

**Figure 4:**
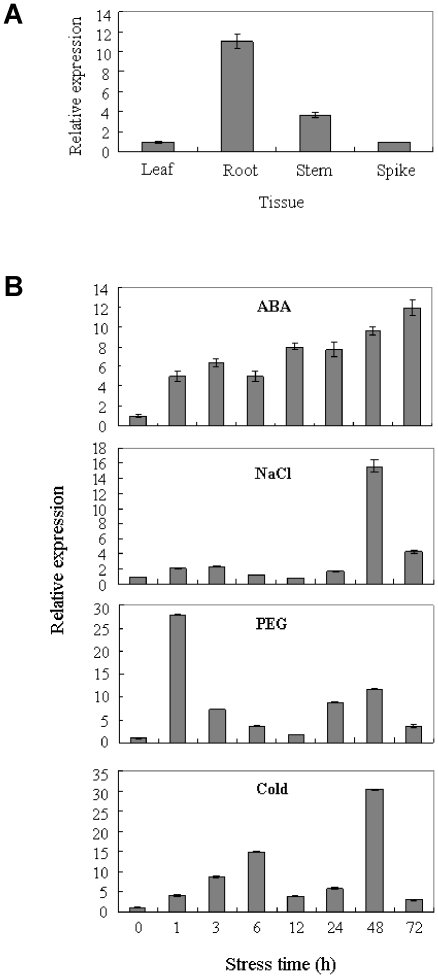
Expression patterns of *TaSnRK2.8* in various tissues and in response to various stresses. (A) Expression patterns of *TaSnRK2.8* in different wheat tissues. (B) Expression patterns of *TaSnRK2.8* under various stress conditions. *Tubulin* was used as an internal control. The vertical column indicates the relative transcript level. Data represent means ±SD of three replicates.

Quantitative real-time PCR were used to analyze the expression patterns of *TaSnRK2.8*. As shown in Figure 4A, *TaSnRK2.8* was constitutively expressed in wheat, strongly in roots, weakly in stems, and marginally in leaves and spikes.

Various up-regulated expression patterns occurred under diverse abiotic stresses (Figure 4B). For ABA treatment, the expression level of *TaSnRK2.8* increased gradually and reached its maximum after 72 h. Under NaCl stress, *TaSnRK2.8* peaked at 48 h, and then decreased. Under PEG stress, the transcription level increased rapidly, peaked at 1 h, and then declined sharply to a lower level. Under cold stress, expression increased gradually for 6 h, declined at 12 and 24 h, and reached a maximum peak at 48 h before declining again at 72 h.

### Identification of transgenic plants

Transgenic plants were firstly screened on kanamycin plates, and then re-confirmed by detection of GFP fluorescence and RT-PCR (Figure S1). Six transgenic lines were randomly selected to detect gene expression levels. The expression levels of *TaSnRK2.8* in different transgenic lines varied significantly (Figure S1).

### Overexpression of *TaSnRK2.8* confers enhanced seedling growth

**Figure 5:**
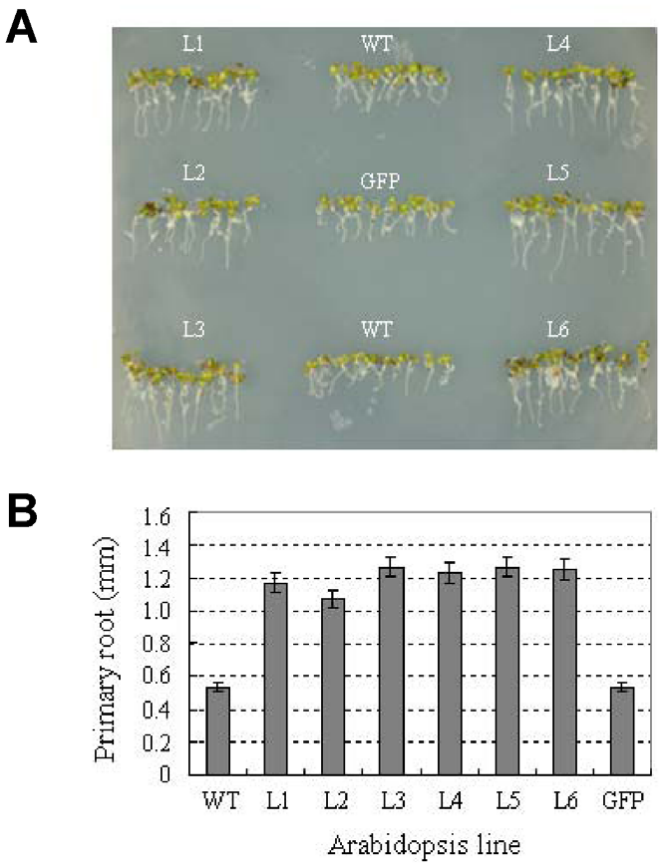
Overexpression of *TaSnRK2.8* enhances root growth. (A) Comparison of root morphologies between *TaSnRK2.8* lines and two controls grown on MS medium for one week. (B) Comparison of primary root lengths. L1–L6, six independent *TaSnRK2.8* transgenic lines; WT, wild type; GFP, *GFP* plants. Values are mean ± SE, n = 10. ** Significantly different from the controls at *P*<0.01 (*F*-test).

To evaluate the effect of *TaSnRK2.8* in transgenic breeding for abiotic stress tolerance, phenotypes of *TaSnRK2.8* plants were characterized at different developmental stages. Morphological assays indicated no differences in seed germination rate and seed production between transgenic and WT plants (Figure S2). However, the primary roots of *TaSnRK2.8* plants were significantly longer than those of the two controls (*P*<0.01) (Figure 5). In addition, the seedlings of *TaSnRK2.8* plants were slightly bigger than the controls, but the differences did not reach significant levels (*P*>0.05), and disappeared within two weeks of culture on MS medium (data not shown). These results were consistent with the function of *SnRK2.8* in *Arabidopsis* [37], suggesting that *TaSnRK2.8* might be involved in regulation of shoot and root growth.

### *TaSnRK2.8* might function in carbohydrate metabolism

**Figure 6:**
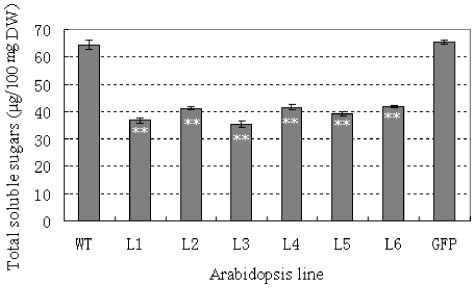
Overexpression of *TaSnRK2.8* leads to significantly decreased total water soluble sugars in *Arabidopsis*. L1–L6, six individual *TaSnRK2.8* lines; WT, wild type; GFP, *GFP* plants. Values are mean ± SE, n = 10. ** Significantly different from the controls at *P*<0.01 (*F*-test).

To investigate the role of *TaSnRK2.8* in carbohydrate metabolism, the total soluble sugars of *TaSnRK2.8* plants were measured. The total soluble carbohydrate of transgenic lines were significantly lower than the WT and *GFP* controls under well-watered conditions (*P*<0.01) (Figure 6), suggesting that *TaSnRK2.8* might be involved in carbohydrate metabolism.

### Physiological characterization of transgenic plants

**Figure 7:**
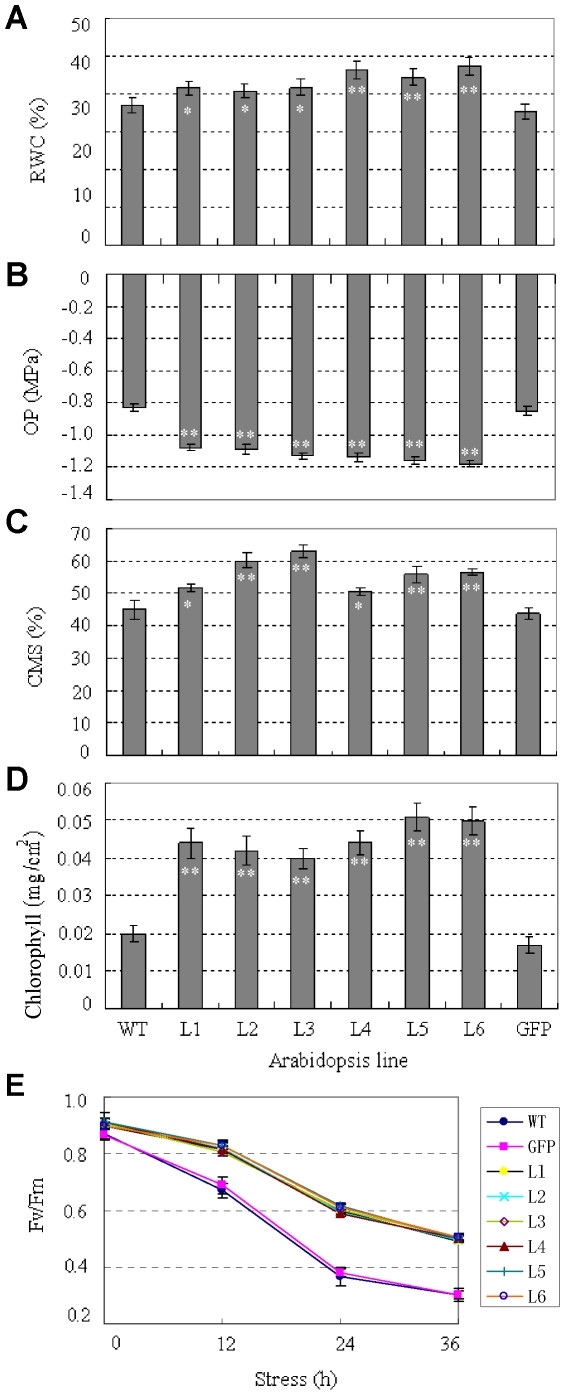
Physiological characterizations of *TaSnRK2.8* plants. (A) Comparison of WRA of detached rosettes of *TaSnRK2.8* plants and WT and *GFP* controls. (B) *TaSnRK2.8* plants had lower OP than controls cultured under well-watered conditions. (C) Comparison of CMS of *TaSnRK2.8* plants and controls after salinity treatment for 5 h. (D) *TaSnRK2.8* plants had significantly more chlorophyll content than controls. (E) Changes in variable to maximum fluorescence ratios (Fv/Fm) in *TaSnRK2.8* plants and WT and *GFP* controls upon high salt stress. L1–L6, six individual *TaSnRK2.8* transgenic lines; WT, wild type; GFP, *GFP* plants. Values are mean ± SE, n = 10. * indicates significant difference between *TaSnRK2.8* plants and WT control with *F*-test (* *P*<0.05, ** *P*<0.01).

Physiological traits related to plant stress tolerance, including WRA, OP, free proline, CMS, chlorophyll content and chlorophyll florescence were analysed. Compared to WT and *GFP* plants, the six transgenic lines showed higher WRA than the two controls (*P*<0.05, *P*<0.01) (Figure 7A). *TaSnRK2.8* plants had significantly lower OP than WT and *GFP* plants(*P*<0.01) (Figure 7B). Free proline is an osmoprotecting molecule, which accumulates in response to water stress and salinity [38]. In the present study, there was no difference in free proline content between WT and transgenic plants (data not shown). Therefore, free proline might not be the reason of the decreased OP.

To identify the cell membrane stability of *TaSnRK2.8* plants under stress, 7-day-old seedlings were treated with NaCl (300 mM) solution on filter paper. After 5 h, WT and *GFP* plants began to wilt, but no signs of stress were evident on *TaSnRK2.8* plants (data not shown). CMS levels in transgenic lines were significantly higher than in the two controls (*P*<0.01), strongly indicating that over-expression of *TaSnRK2.8* increased CMS of *Arabidopsis* under salt stress (Figure 7C).

Under normal conditions, leaves of *TaSnRK2.8* plants were slightly more green than WT and *GFP* plants (data not shown), and *TaSnRK2.8* plants had significantly higher chlorophyll contents than the controls (*P*<0.01) (Figure 7D). Additionally, photochemical efficiency of PSII (Fv/Fm) determinations showed that chlorophyll florescence was slightly higher in *TaSnRK2.8* plants than in controls under normal conditions, but the difference was not significant (*P*>0.05) (Figure 7E). Under salt stress, the chlorophyll florescence decreased by approximately 10% in *TaSnRK2.8* plants and 23% in the two controls at 12 h. With additional time, the Fv/Fm for the control plants decreased much faster than in *TaSnRK2.8* plants (Figure 7E). These results clearly showed that *TaSnRK2.8* plants had a more robust photosynthetic potential.

### Overexpression of *TaSnRK2.8* results in enhanced drought, salt and cold tolerances

**Figure 8:**
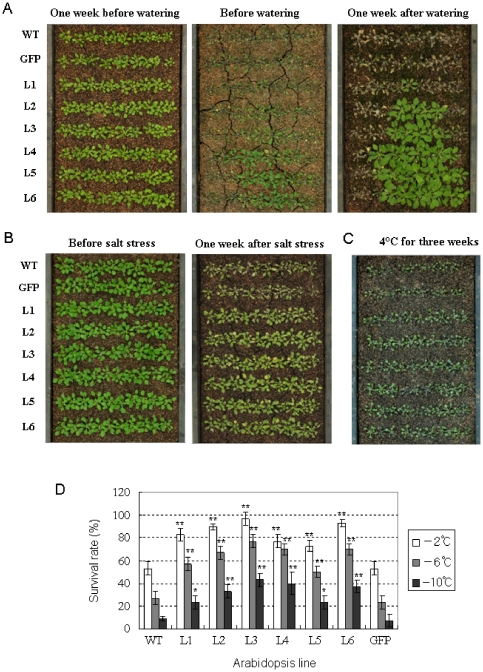
*TaSnRK2.8* plants shows improved stress tolerance. (A) *TaSnRK2.8* plants and controls grown under drought stress. After planting in soil, seedlings were withheld from water for two weeks, and then re-watered for one week. (B) *TaSnRK2.8* plants and controls under salt stress. Two-week-old seedlings were treated with 300 mM NaCl. (C) *TaSnRK2.8* plants and controls cultured at 4°C. (D) Survival rates (%) under freezing conditions were determined as the number of visibly green plants after rehydration. Values are mean ± SE, n = 10. * indicates significant difference between *TaSnRK2.8* plants and WT control with *F*-test (* *P*<0.05, ** *P*<0.01).

To examine the roles of *TaSnRK2.8* in plant stress responses, *TaSnRK2.8* lines and control plants were exposed to various abiotic stresses. After a two-week-water-withholding treatment, the rosette leaves of WT and *GFP* controls wilted severely and most became darker and died. By comparison, only some of the *TaSnRK2.8* plants were slightly wilted. After re-watering for one week, all the control plants were dead, whereas 10–80% of *TaSnRK2.8* plants had survived (Figure 8A). One week after salinity treatment, almost all control plants had died, whereas *TaSnRK2.8* plants were still green, clear evidence that the transgenic plants were much more tolerant than the controls (Figure 8B). For cold stress analysis, the plants were cultured at 4°C. Three weeks later, differences in seedling size became evident, and *TaSnRK2.8* plants were larger than the controls (Figure 8C). Further freezing tolerance assays showed that survival rates of *TaSnRK2.8* plants under −2°C, −6°C and −10°C were significantly more than the controls (Figure 8D), suggesting that *TaSnRK2.8* plants have increased tolerance to freezing stress. These results indicated that overexpression of *TaSnRK2.8* conferred enhanced tolerance to drought, salt and cold stresses in *Arabidopsis*.

### Expression pattern of stress-responsive genes in *TaSnRK2.8* plants

**Figure 9:**
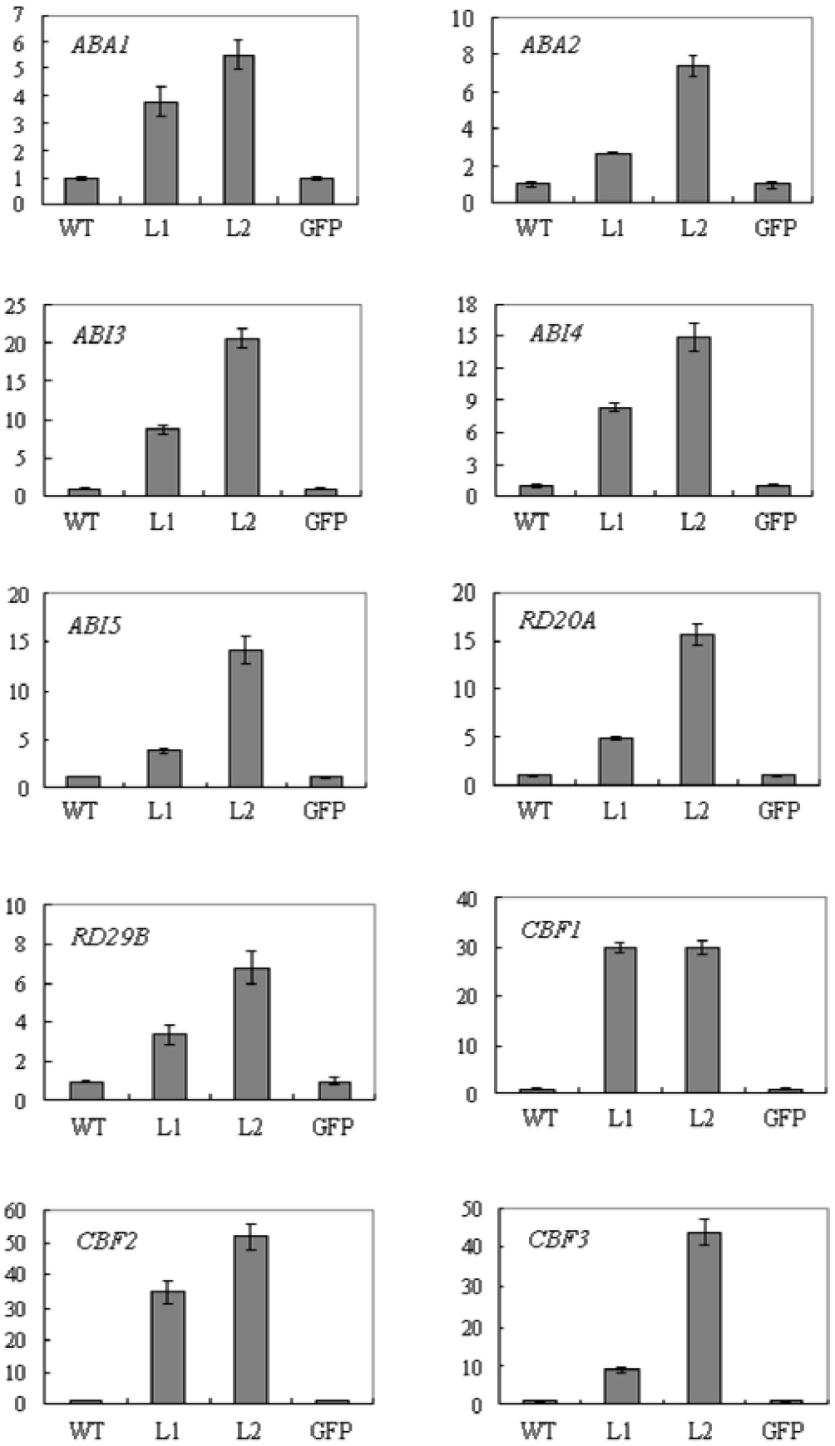
Comparisons of the relative transcript levels of *ABA1*, *ABA2*, *ABI3*, *ABI4*, *ABI5*, *RD20A*, *RD29B*, *CBF1*, *CBF2* and *CBF3* in *TaSnRK2.8* plants and WT/*GFP* control under normal conditions. *Actin* was used as an internal control. The vertical column indicates the relative transcript level. Data represent means ±SD of three replicates.

Expression pattern analyses in wheat revealed that *TaSnRK2.8* was involved in response to PEG, NaCl and cold stresses and ABA application. To elucidate the molecular mechanism of *TaSnRK2.8* in stress response, the expression levels of the genes functioning in ABA biosynthesis and signaling or those involved in stress protection were investigated in *TaSnRK2.8* plants. As expected, under normal conditions, the expressions of *ABA1, ABA2, ABI3, ABI4, ABI5, CBF1, CBF2, CBF3, RD20A, RD29B* were found to be consistently and significantly higher in *TaSnRK2.8* plants than in WT/*GFP* plants, whereas there was no significant induction of expression of *ABI1, ABI2, COR15A* in both *TaSnRK2.8* and WT/*GFP* plants (Fig. 9). Under salt stress, there was the same trend (data not shown).

## Discussion

In this study, a dehydration-inducible cDNA library of wheat was screened for transcripts that might be significantly up-regulated under PEG stress. The identified protein kinase gene, designated as *TaSnRK2.8*, was cloned and characterized. The deduced amino acid sequence shows high homology with counterpart SnRK2 family members from rice, maize and *Arabidopsis*, implying the occurrence of *SnRK2.8* before separation of monocots and dicots. Furthermore, phylogenetic tree analysis revealed that *TaSnRK2.8* is a typical subclass III SnRK2 subfamily member.

N-terminal myristoylation and transmembrane spanning regions are essential for proteins to function in mediating membrane targeting and signal transduction in plant responses to environmental stress [39], [40]. In the catalytic domain of TaSnRK2.8, one potential N-myristoylation site and one potential transmembrane spanning region were identified, strongly suggesting that TaSnRK2.8 may interact with the cell-membrane system when responding to stress. In yeast, SNF1 kinase was localized to the nucleus, vacuole, and cytoplasm [41], and was involved in signal transduction pathways by interacting with RNA polymerase II holoenzyme to activate transcription of glucose-responsive genes [42]. In the present study, the presence of TaSnRK2.8 in the cell membrane, cytoplasm and nucleus suggested that TaSnRK2.8 might have different functions in wheat. The results were confirmed through observing TaSnRK2.8-GFP-*Arabidodsis* (Figure S1), and detailed location and the signal transduction pathway of *TaSnRK2.8* in stressed plants remain to be determined.

Numerous studies demonstrate that *SnRK2.8* genes are involved in response to multi-environmental stresses [20], [24], [43]. In this study, *TaSnRK2.8* was induced not only by NaCl, PEG and cold, but also by ABA (Figure 4B), suggesting that *TaSnRK2.8* was involved in an intricate network for multi-environmental stress responses. Comparing these expression patterns, induction of *TaSnRK2.8* by PEG was more rapid than induction by ABA. This could suggest that factors other than ABA might also involve in *TaSnRK2.8* induction, and/or that ABA does not induce *TaSnRK2.8* directly. In addition, the response of *TaSnRK2.8* under water deficit stress is much faster than to NaCl, cold and ABA treatment. These differences also occurred at the expression level, suggesting that *TaSnRK2.8* is more sensitive to drought stress.

To assess the feasibility of using *TaSnRK2.8* in transgenic breeding, phenotypic traits of *TaSnRK2.8* plants were closely monitored throughout the entire growth cycle (Figure S2). The results clearly demonstrated that *TaSnRK2.8*-overexpression does not retard plant growth, whereas it improved the root growth of transgenic plants (Figure 5A), which could benefit the uptake of water and nutrients under osmotic stress conditions.

Mammalian AMPK and yeast SNF1 act as energy-level sensors that function to regulate metabolism during low-energy conditions [44], [45]. Compelling evidence indicates that plant SnRK1 and SnRK3 proteins have roles in regulating energy metabolism and stress signal transduction [11], [46]. Until now, there has limit reports of SnRK2 function in carbohydrate metabolism. Recently, SnRK2.6 protein, clustered with TaSnRK2.8 in the same clade, subclass III (Figure 2), was found to mediate the regulation of sucrose metabolism and plant growth in *Arabidopsis* [47]. In the present study, overexpression of *TaSnRK2.8* led to significantly decreased total soluble sugar content in *Arabidopsis* (Figure 6), suggesting that *TaSnRK2.8* might function in carbohydrate metabolism. In further research, more effort should be given to deciphering the signals and molecular mechanisms of *TaSnRK2.8* in carbohydrate metabolism, especially in regard to crucial downstream substrates.

Physiological indices, including WRA, CMS, OP and free proline, are typical physiological parameters for evaluating abiotic stress tolerance and resistance in crop plants. To maintain a stable intracellular environment in the presence of external environmental stresses, many plants decrease their cellular osmotic potentials through accumulation of intracellular organic osmolytes such as proline, total soluble sugar contents, glycine betaine and mannitol [48]–[50]. In our study, *TaSnRK2.8* plants had significantly lower OP than controls (Figure 7B), but the free proline was not significantly increased, suggesting that free proline might not account for osmolyte augmentation. In addition, *TaSnRK2.8* plants had significantly lower total soluble sugar contents than controls under well-watered conditions (Figure 5), but the difference vanished very soon after exposesure to salt stress (data not shown). Therefore, total soluble sugar content was also not a cause of osmolyte augmentation. The results of WRA and CMS determinations were consistent with the OP results, and suggest that the enhanced multi-stress tolerance might be due to osmolyte augmentation. Thus, other types of osmolytes might be attributed to the enhanced OP in *TaSnRK2.8* plants.

Chlorophyll fluorescence from intact leaves, especially fluorescence induction patterns, is a reliable, non-invasive method for monitoring photosynthetic events and reflects the physiological status of plants [51]. The maximum efficiency of PSII photochemistry, measured as Fv/Fm, is an direct reflection of the PSII activity, and environmental stresses are associated with decreased Fv/Fm ratio [52]. In this study, we observed smaller decreases in Fv/Fm ratios in *TaSnRK2.8* plants under salt stress (Figure 7E), and witnessed significantly higher chlorophyll contents in transformants. Thus *TaSnRK2.8* plants might have more robust photosynthetic capabilities than non-transformed controls.

Based on the phenotypic and physiological characteristics of *TaSnRK2.8* plants, we speculate that the enhanced multi-stress tolerance is possibly due to an improved root system, increased capability of osmotic adjustment, and robust photosynthetic capabilities. Under osmotic stress, longer roots might facilitate *TaSnRK2.8* plants to absorb more water. Robust photosynthetic capability might help transgenic plants to become more vigorous, and increased osmolytes might be helpful in reducing water loss and maintaining a higher WRA in plant cells, thus leading to enhanced water retention ability, benefiting the maintenance of regular cell turgor and avoiding damage to cell membranes, consequently enhancing drought tolerance. Under cold stress, lower OP commonly means more solutes in the plant sap, resulting in lower freezing points and hence reduced cold damage.

Under abiotic stresses, ABA is often recruited as the primary signal for increasing the transcription levels of the stress responsive genes. The function of *ABA1, ABA2* and *ABA3* genes in ABA biosynthesis has been well established [53], [54], and the most intensively investigated regulators of ABA signaling include several *ABI* genes, of which *ABI1* and *ABI2* are negative regulators, and *ABI3*, *ABI4* and *ABI5* regulate ABA responses positively [55]–[58]. In the present study, the expressions of *ABA1, ABA2*, and ABA positive regulators (*ABI3*, *ABI4* and *ABI5*) in *TaSnRK2.8* plants were substantially increased compared to WT plants. Therefore, it was indicated that the enhanced stress tolerance conferred by *TaSnRK2.8* overexpression may be attributed to increased ABA biosynthesis and signaling, which result in greater expression of several stress responsive genes. Moreover, it is well known that CBF genes are mainly involved in ABA independent regulation of stress responsive genes [59]. Here, the transcript levels of *CBF1*, *CBF2* and *CBF3* were also significantly higher in *TaSnRK2.8* plants than the controls, suggesting that there may be ABA independent stress signaling pathways involved in *TaSnRK2.8*-mediated stress tolerance. Meanwhile, the high transcript levels of stress responsive genes in *TaSnRK2.8* plants suggest that *TaSnRK2.8* may act upstream of these genes in stress tolerance and is therefore involved in a crosstalk between ABA-dependent and ABA-independent signaling networks.

## Supporting Information

Figure S1 **Identification of the *TaSnRK2.8* transformed *Arabidopsis* plants.** (A) Determination of green fluorescence in roots of transgenic *Arabidopsis* plants. Assays were performed at the seedling stage with a laser‐scanning confocal microscope. The images were taken in dark field for green fluorescence, and the root outline and combination are in bright field. (B) RT‐PCR analysis of transgenic plants. M: 200‐bp ladder; Lane 1, p35S‐*TaSnRK2.8*‐*GFP*‐NOS plasmid DNA (positive control); Lane 2, wild‐type *Arabidopsis* (negative control); Lanes 3‐19, p35S‐*TaSnRK2.8*‐*GFP*‐NOS transformed plants. (C) Expression levels of *TaSnRK2.8* in transgenic *Arabidopsis* lines L1‐L6. The lowest expression of *TaSnRK2.8* in L1 was regarded as standard.
(TIF)

Figure S2 **Morphological characterization of *TaSnRK2.8* plants.** (A) Comparison of seed germination and seedlings between *TaSnRK2.8* transformants and controls grown on MS medium. (B) Phenotypes of mature transgenic lines and WT grown in soil for four weeks. (C) Grain yields of *TaSnRK2.8* and WT plants. The seeds of transgenic *TaSnRK2.8* and WT plants cultured under well‐watered conditions were harvested separately. The grain yield of each plant was measured after dehydration, and there was no significant difference. L1–L6, six individual *TaSnRK2.8* transgenic lines; WT, wild type; GFP, *GFP* transgenic line. Values are mean ± SE, n=10.
(TIF)
