# Supplementary figures and images for: Overexpression of a Common Wheat Gene TaSnRK2.8 Enhances Tolerance to Drought, Salt and Low Temperature in Arabidopsis
Source: PLoS One. 2010 Dec 30;5(12):e16041. doi: 10.1371/journal.pone.0016041 (PMC3012728; doi:10.1371/journal.pone.0016041)

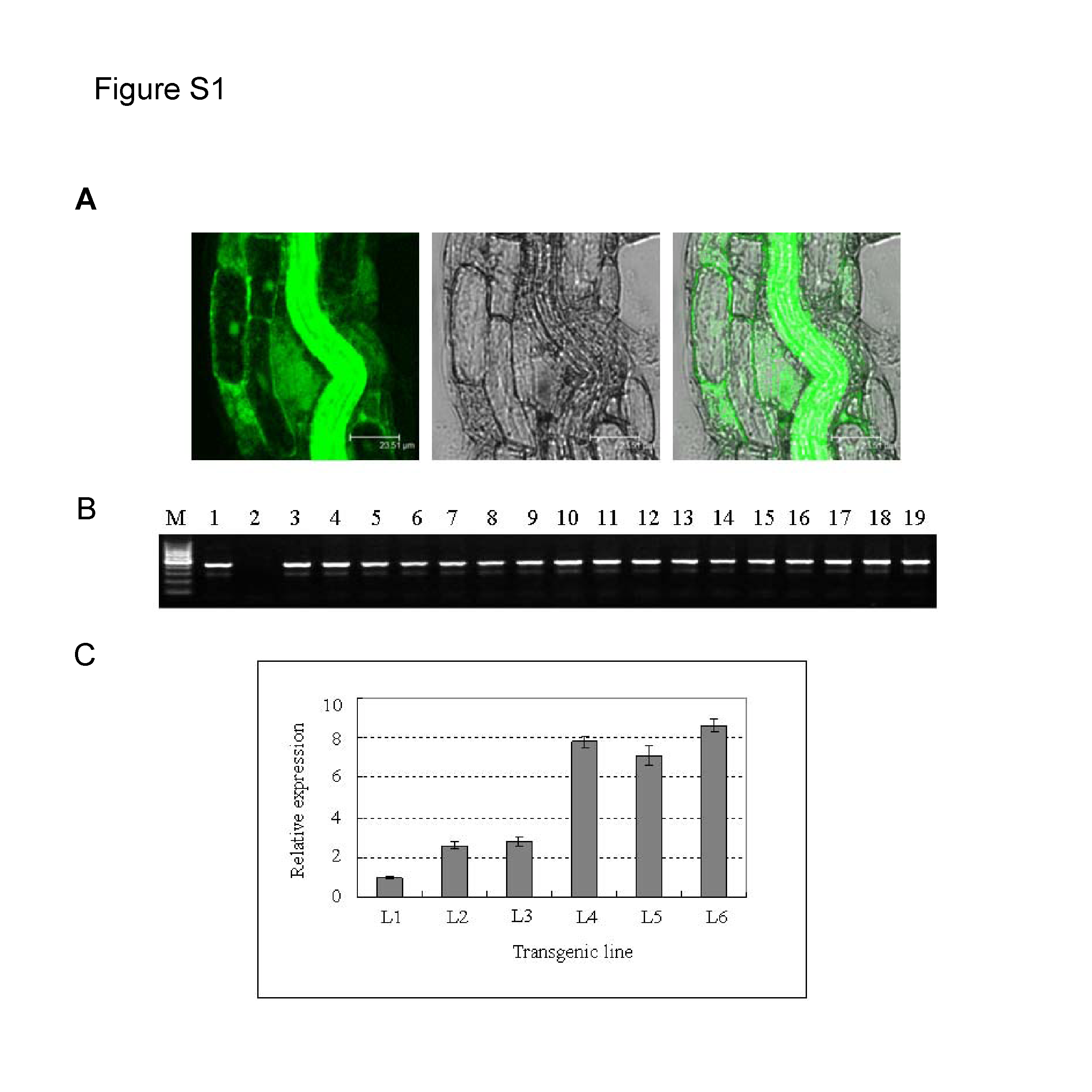

Supplement: Figure S1 — Identification of the TaSnRK2.8 transformed Arabidopsis plants. (A) Determination of green fluorescence in roots of transgenic Arabidopsis plants. Assays were performed at the seedling stage with a laser‐scanning confocal microscope. The images were taken in dark field for green fluorescence, and the root outline and combination are in bright field. (B) RT‐PCR analysis of transgenic plants. M: 200‐bp ladder; Lane 1, p35S‐TaSnRK2.8‐GFP‐NOS plasmid DNA (positive control); Lane 2, wild‐type Arabidopsis (negative control); Lanes 3‐19, p35S‐TaSnRK2.8‐GFP‐NOS transformed plants. (C) Expression levels of TaSnRK2.8 in transgenic Arabidopsis lines L1‐L6. The lowest expression of TaSnRK2.8 in L1 was regarded as standard. (TIF) [file pone.0016041.s001.tif]

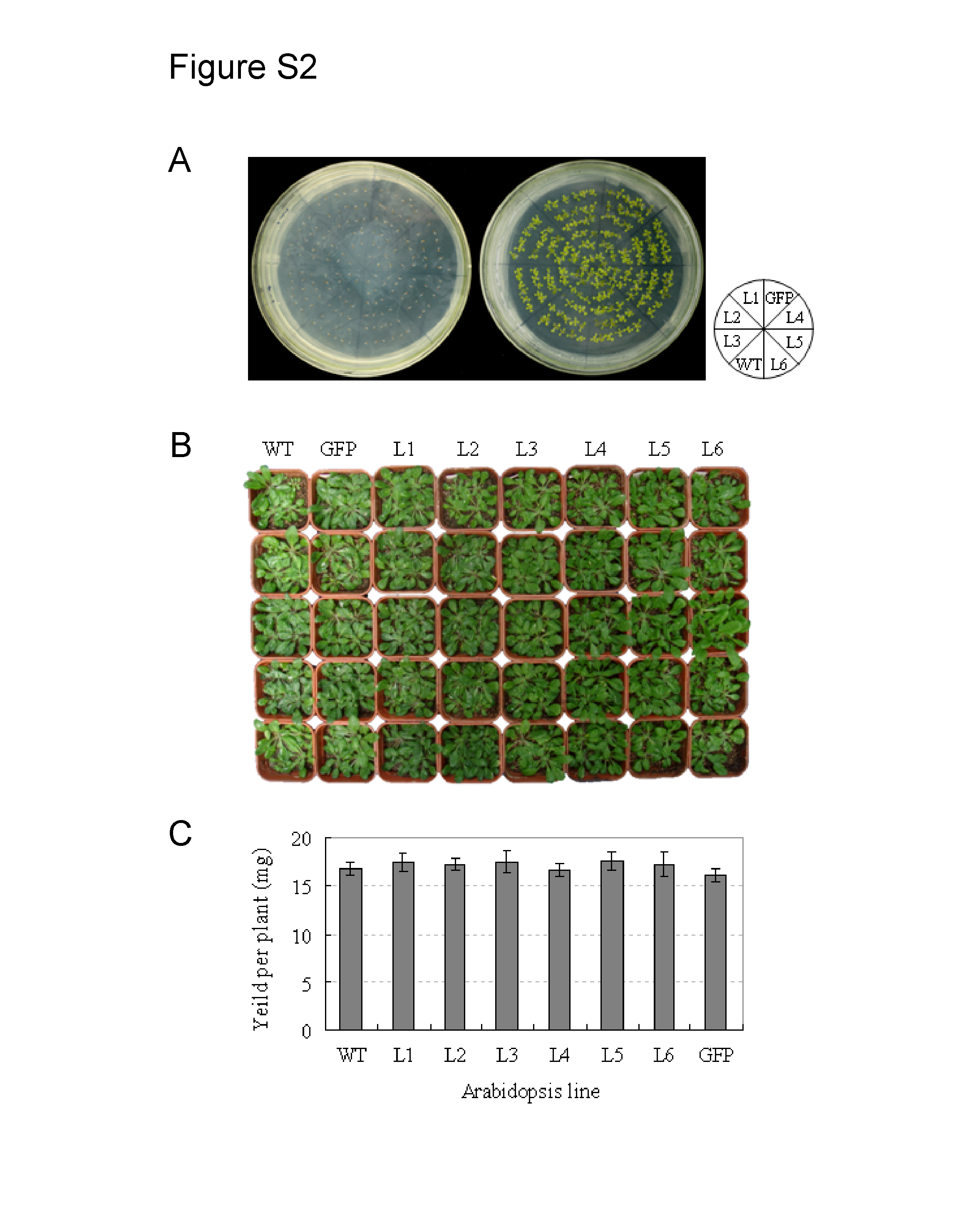

Supplement: Figure S2 — Morphological characterization of TaSnRK2.8 plants. (A) Comparison of seed germination and seedlings between TaSnRK2.8 transformants and controls grown on MS medium. (B) Phenotypes of mature transgenic lines and WT grown in soil for four weeks. (C) Grain yields of TaSnRK2.8 and WT plants. The seeds of transgenic TaSnRK2.8 and WT plants cultured under well‐watered conditions were harvested separately. The grain yield of each plant was measured after dehydration, and there was no significant difference. L1–L6, six individual TaSnRK2.8 transgenic lines; WT, wild type; GFP, GFP transgenic line. Values are mean ± SE, n=10. (TIF) [file pone.0016041.s002.tif]
